# Edaravone-Encapsulated Agonistic Micelles Rescue Ischemic Brain Tissue by Tuning Blood-Brain Barrier Permeability: Erratum

**DOI:** 10.7150/thno.108498

**Published:** 2025-01-09

**Authors:** Qu Jin, Yu Cai, Sihan Li, Haoran Liu, Xingyu Zhou, Chunqiang Lu, Xihui Gao, Jun Qian, Jun Zhang, Shenghong Ju, Cong Li

**Affiliations:** 1 Key Laboratory of Smart Drug Delivery, Ministry of Education, School of Pharmacy, Fudan University, 826 Zhangheng Road, Shanghai 201203, P. R. China.; 2 Jiangsu Key Laboratory of Molecular and Functional Imaging Department of Radiology, Zhongda Hospital, Medical School of Southeast University, 87 Dingjiaqiao Road, Nanjing 210009, P. R. China.; 3 Department of Radiology, Huashan Hospital, Fudan University, 12 Wulumuqi Middle Road, Shanghai 200041, P. R. China.

The authors regret that the original version of our paper, unfortunately, contained incorrect pictures in Figure 3D and Figure 5E. In Figure 3D, the fluorescence images of the EDV-AM group (4 h) were inadvertently duplicated from the free EDV group (12 h). In Figure 5E, the fluorescence image of the ischemic brain striatum of the PBS group, stained with the Tunnel kit, was inadvertently mislaid. The correct figures are shown below. The authors confirm that these corrections do not change the results or conclusions of the article. The authors are deeply sorry and sincerely apologize for any inconvenience or misunderstanding that may have caused.

## Figures and Tables

**Figure 3:**
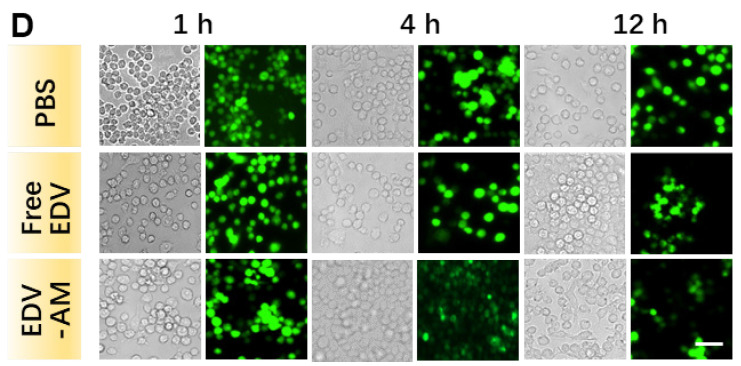
Corrected image for the original Figure 3D. White light and fluorescence images of OGD-treated live RAW264.7 macrophage cells at selected time-points post addition of PBS, free EDV, or EDV-AM.

**Figure 5:**
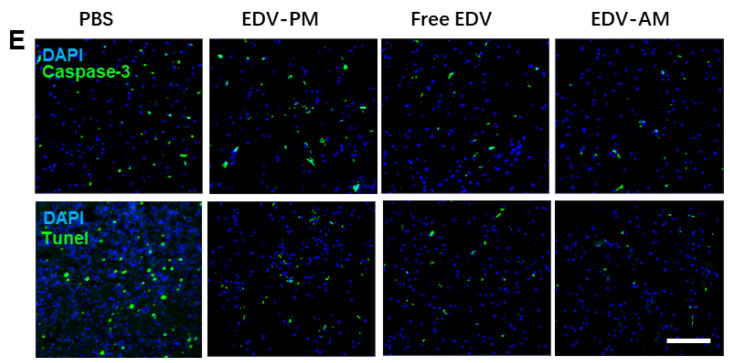
Corrected image for the original Figure 5E. Fluorescence microscopic images of ischemic brain striatum stained by Tunnel kit or immunostained by caspase-3 antibody at 14 days post treatments.

